# A retail investor in a cobweb of social networks

**DOI:** 10.1371/journal.pone.0276924

**Published:** 2022-12-30

**Authors:** Tamara Teplova, Aleksandr Tomtosov, Tatiana Sokolova

**Affiliations:** 1 Centre for Financial Research & Data Analytics, Faculty of Economic Sciences, HSE University, Moscow, Russia; 2 Faculty of Economic Sciences, HSE University, Moscow, Russia; URV: Universitat Rovira i Virgili, SPAIN

## Abstract

In this study, using AI, we empirically examine the irrational behaviour, specifically attention-driven trading and emotion-driven trading such as consensus trading, of retail investors in an emerging stock market. We used a neural network to assess the tone of messages on social media platforms and proposed a novel Hype indicator that integrates metrics of investor attention and sentiment. The sample of messages, which are written in Russian with slang expressions, was retrieved from a unique dataset of social network communication of investors in the Russian stock market. Applying different portfolio designs, we evaluated the effectiveness of the new Hype indicator against the factors of momentum, volatility, and trading volume. We found the possibility of building a profitable trading strategy based on the Hype indicator over a 6-month time horizon. Over short periods, the Hype indicator allows investors to earn more by buying stocks of large companies, and over «longer» periods, this indicator tends to perform better for illiquid stocks of small companies. As consensus trading tends to produce negative returns, the investment strategy of ‘Go against the crowd’ proves rewarding in the medium term of 3 months.

## Introduction

In recent years, a number of factors have contributed to a massive inflow of retail cash into the stock market. These factors include easy monetary policy of many central banks, falling rates on debt instruments and bank deposits, COVID-19-related fiscal relief packages, and the development of investment platforms designed for trading via mobile apps and for discussion in social networks. Stock exchanges globally have experienced an increase in the share of retail investors in trading volume [1]. For example, in Russia, the share of retail investors in the trading volume of domestic stocks increased from 34% in 2013 to 40% in 2020, and it reached 43% in mid-2021 ([1]; MOEX bulletins). In the largest stock market of the US, the share of retail investors increased from 13% in 2013 to 24% in mid-2021 (source: Bloomberg Intelligence) (https://www.bloomberg.com/news/articles/2021-11-17/retail-traders-retreat-as-choppy-markets-challenge-easy-profits). This increase in the share of retail investors in the stock market has led to the emergence of so-called ‘meme stocks’ and a strategy of buying stocks of the most discussed companies [2, 3]. US and Russian regulatory authorities have expressed concerns about the activity of retail investors in social networks and the possibility of their collective actions to drive stock prices. Major hedge funds have begun to identify the most talked-about companies in the Internet to protect themselves from coordinated attacks of non-professional investors (https://www.ft.com/content/04477ee8-0af2-4f0f-a331-2987444892c3).

Our motivation stems from interest in the visibility hypothesis and in a wide range of proposed metrics of investor attention and sentiment. We examined how attention-grabbing stocks and stocks discussed in a certain tone make investors earn or lose money. Many studies have revealed signs of attention-driven buying when more ‘visible’ stocks (e.g., those included in Warren Buffett’s portfolio, a stock index built on President Trump’s Twitter account, etc.) attract a great deal of investor attention and this significantly impacts the stock returns [4–7]. [8] constructed a news-based manager sentiment based on the tone of managers’ news reports and conclude that it can predict excellently the subsequent month return. Its ability to predict stock returns is higher during the periods of optimistic mood.

At the same time, the impact of messages posted by retail investors in social networks is a less studied question. We fill this gap by analysing a number of metrics: 1) metrics of attention based on trading characteristics (i.e., technical analysis) and messages on social media, and 2) metrics of investor sentiment that capture the tone of messages, in particular, a positive consensus perception of a company. Our novel Hype indicator integrates social attention and the tone of messages and allows us to analyse the role of consensus perception of a stock (unanimously positive perception), which is closely related to the recently popular phenomenon of ‘meme stocks’ [2].

A traditional point of view regarding the determinants of asset pricing and the portfolio construction is the emphasis on financial indicators and macro indicators [9–11]. In recent years, investor communication channels have been transformed. Now an important characteristic influencing price behavior is the opinion of retail investors in social networks and instant messengers [2, 12]. [10] proposed a theoretical model in which noise traders indeed earn higher expected returns than the rational ones. So, the first research question is the impact of social communication (discussions on investment topics) of retail investors on asset pricing behavior and the opportunity of building a profitable strategy.

Based on discussions of companies in social networks, two types of metrics can be distinguished: characteristics of sentiment (tonality of messages) of investors and characteristics of attention [13-15]. The first research in this area was mainly devoted to the analysis of attention: the number of search queries on the Internet was considered, the frequency of mentions of companies [15, 16]. Further, the direction of sentiment analysis began to actively develop [13, 14]. Sentiment research became possible with the use of artificial intelligence (AI) techniques. Much progress has been made in terms of the analysis of texts in English (for example, the neural network Google BERT), while the analysis of texts in the languages of developing countries is still in its beginning [17]. The analysis of texts on investment topics in specific languages, using slang, requires development, which motivates our study. We classify by tonality the texts in Russian published on forums and in messengers with the use of AI. So, the scope of research questions is the following. How different are the two types of metrics–characteristics of sentiment and attention—in terms of asset price reactions and investor earnings? Are characteristics of sentiment and attention more important for small companies than large companies in terms of the ability to build profitable strategies? On what time horizon do sentiment characteristics allow an investor to build profitable strategies?

There are manipulations in the markets when at the same time there is a massive implicit advertising of securities by brokers, issuers themselves, and investors. Investors should be concerned about this. A surge in the number of messages devoted to a company may be the result of manipulation or, conversely, indicate real changes in the company’s fundamentals. Accordingly, the final research question is: should an investor beware of following the opinion of the crowd?

Due to large volumes of information, irrational investors tend to focus on ‘obvious’ data rather than on detailed data such as fundamental indicators. [4] used stocks’ trading characteristics (such as abnormal trading volume and extreme returns) and a company’s mentions in the news as proxies for investor attention. However, these proxies are far from perfect since they only indirectly measure investor attention. For example, posting a news item does not necessarily imply that investors will read and remember it.

The most recent papers have recognized the important role of investor sentiment and confirmed its impact on stock prices [18–20].

The main objective of our study is to utilize the analysis of attention and sentiment in social networks to detect the existence of behavioural biases in a low-liquidity, non-English speaking emerging market and to test their persistence. We use artificial intelligence (AI) for performing textual analysis. A significant novelty in our research is the use of a unique dataset and comprehensive coverage of online written communication among Russian retail investors who are presently registered holders of approximately 43% of active brokerage accounts on the Moscow Exchange.

With this paper, we make four contributions to the literature that analyses investor attention and sentiment. First, we advance a novel Hype indicator, which is capable of capturing the impact of attention-driven and emotion-driven trading. For each company, the Hype indicator takes into account the intensity of discussion (i.e., the frequency of the company’s mentions on social media) and a positive tone of messages. Previous works only considered attention and sentiment metrics separately [13, 21–23]. It should be noted that our Hype indicator cannot fully identify ‘meme stocks’, but it may be used as a complement to other tools for identification.

Second, we composed various portfolios and compared their performance with respect to a wide range of characteristics: the Hype indicator, sentiment metrics with social networks as the source material, attention metrics with social networks as the source material, and traditional metrics of investor attention derived from stocks’ trading characteristics. We modify the conventional approach by using two data sources: 1) the MFD platform with exclusively finance-related thematic forums, which is popular among people over 30 years old, and 2) Telegram, which has a variety of channels that are most popular among Millennials. One interesting detail that differentiates these two platforms is that MFD has always been informally approved by Russian authorities but this is not the case with Telegram. During the period from 2018 to the first half of 2020, Telegram was not considered legitimate in Russia, and was officially banned from operating within the country, but de facto, it continued to operate relatively normally despite numerous IP blockades. Only in June 2020 did the Russian authorities officially permit investors to use this social media platform (https://www.reuters.com/article/us-russia-telegram-ban-idUSKBN23P2FT).

Third, we built a unique neural network that processes investors’ communications as messages rather than as separate words. While newspapers mostly use formal language and content can be categorized as positive, neutral, or negative with the help of available dictionaries and software packages, communication on social media platforms often uses informal language and many slang expressions (for example, MMC Norilsk Nickel is frequently referred to as ‘mink’ or ‘hammock’). The task of tone categorization is additionally complicated by the fact that slang language evolves over time and by the simultaneous presence of negative and positive words in one message (for example, “the market is falling, but we will still grow”). Therefore, we forwent the idea of using available dictionaries and switched to building a self-learning neural network. [24] highlight the issue of the effectiveness of conventional vocabularies in explaining financial statements. The authors refer to the use of the Harvard IV-4 Psychosociological Dictionary in [25].

We manually labelled over 30,000 messages and compiled a dictionary of Russian trading slang words and expressions. We applied a CNN-based model following [13, 14].

Fourth, some previous studies [13, 14, 23] assumed an identical impact of sentiment on all stocks. In contrast, we believe that the effectiveness of portfolios, composed of the basis attention and sentiment metrics, may differ for blue-chip stocks for which there is a high proportion of non-residents and professional investors among holders, stocks of mid-sized companies, and stocks of small companies that are primarily held by domestic retail investors.

As our major research findings, we confirmed the presence of the visibility effect in the Russian stock market. Investor attention and sentiment, revealed by the content analysis of social network communication, are significant characteristics in asset pricing along with traditional indicators such as price momentum, volatility, and trading volume. Over short periods, the Hype indicator allows investors to earn more by buying stocks of large companies, and over longer periods, the indicator tends to perform better for illiquid stocks of small companies. As consensus trading tends to produce negative returns, the investment strategy of ‘Go against the crowd’ proves rewarding in the medium term of 3 months. Thus, one negative side of social networks is that they facilitate the formation of a consensus, which further governs the actions of their users (retail investors). This potentially poses a risk of price manipulation and especially large losses for holders of illiquid stocks.

The remainder of the paper is structured as follows. Section 2 reviews previous research on behavioural biases in the stock market and on the measurement of sentiment and attention of retail investors. Section 3 describes the sample. Section 4 presents the neural network-based textual processing algorithm and the methodology of constructing attention and sentiment indicators and portfolio designs. Sections 5 lays out the empirical results. Section 6 presents the robustness check. In the concluding section, we summarize and discuss our findings.

### Literature review and the hypotheses of the study

Investor attention was first proxied by stocks’ trading characteristics [26, 27]. There is no unambiguous understanding of whether high trading volume is a significant factor in explaining stock returns [28]. Some papers [27, 29] reported a positive relationship between high trading volume and stock returns, while other works [30, 31] documented a negative relationship. [31] found a high-volume return discount for small-cap stocks in the Chinese stock market. Such stocks tend to produce relatively low returns following a short period of substantial overpricing. [28] assessed the predictive power of trading volume for short‐term reversals in stock returns on the US and South Korean markets. [29] examined the role of trading volume and volatility in the Russian stock market. [32] measured the investor sentiment by different metrics of volatility: the standard deviation, realized volatility, Parkinson’s estimator and Garman and Klass’s estimator. The author revealed that realized volatility is the only investor sentiment indicator that significantly impacts on stock returns with the emergence of COVID-19 in the EU and US markets.

Later, researchers proposed more advanced proxies for investor attention based on web search queries and news mentions [4, 15]. [33] discovered that negative news has the strongest effect on the cross-sectional stock returns in the Chinese market. The authors found that portfolios constructed by buying stocks without negative news and selling stock with high negative news demonstrate the highest abnormal return, and the excess return exists for more than three months holding periods. [34] concluded that news sentiment does not significantly determine future stock returns, while the sentiment index based on different metrics allows an investor to construct a profitable investment strategy.

A large body of research uses survey data and consumer sentiment indices as sentiment proxies [35]. The most recent attention metrics are based on the analysis of messages posted by retail investors in social networks. Self-isolation restrictions induced by the COVID-19 pandemic contributed to the emergence of impulsive investor behaviour such as ‘meme stock’ trading, thereby giving rise to a new direction of research—the implications of ‘meme stock’ trading for asset pricing [2]. [36] considered the metrics based on the tonality and the volume of discussions of companies in Twitter for the Tunisian market. They revealed that sentiment and attention impact the stock returns, but the Granger causality measure is low.

Questions that still remain only partially answered are the automated categorization of the tone of investors’ messages and the measurement of sentiment. Attempts have been made to develop sentiment proxies for the overall stock market [22] and for individual stocks [23].

Previous works focused on broad market stock indices, ETFs, and highly liquid stocks [13, 14, 23, 25]. Only a few papers [14] differentiated companies by size or stock liquidity. [37] concluded that company characteristics, including size, affect the relationship between investor sentiment and stock returns in the South Korean market. [38] concluded that the higher the shareholding ratio of institutional investors in listed companies, the more stable the stock price. Some papers [16, 39] used intraday frequency to analyse the impact of investor attention on stock prices.

One of the most challenging issues in assessing investor sentiment appears to be the development of methods to identify the tone of messages. [21] used official dictionaries, but this method left out slang, emoji icons, and other images that are popular among investors. To address this issue, artificial intelligence techniques can be applied as an alternative to dictionaries. Modern approaches to machine learning for assessing sentiment (primarily to predict stock returns) are given by [13, 14, 23]. [40] showed that the sentiment analysis on natural language sentences can improve forecasting of stock returns. The authors based on the BERT model revealed that taking into account news sentiment allows an investor to construct a portfolio with a higher Sharpe ratio. [41] proposed a generalized Deep Learning-based classification framework for the stock market sentiment analysis and evaluated the performance of different techniques of classification of texts in social networks. They came to conclusion that Convolution neural network (CNN) obtains the best results when dealing with complex data inputs. In our paper, we use CNN-Transformer approach.

The vast majority of papers that analyse the role of investor sentiment in asset pricing focus on the US market [13, 21, 23, 25, 42]. Stock trading in the US is under scrutiny in many countries and discussed in many languages. Consequently, analysing only one online discussion forum results in limited input for textual analysis. In contrast, discussions of trading and investment strategies in the Russian market are carried out on a limited number of social media platforms and almost entirely in the Russian language. This provides an opportunity to analyse a larger share of messages posted by retail investors. [15] constructed a retail investor sentiment index from communication content on Eastmoney, which is the largest Chinese finance-related forum. The authors showed that the constructed index is a statistically significant characteristic for trading volumes, but they did not check if the index might exercise influence over stock prices.

We identified several approaches to understanding whether sentiment is a significant characteristic: 1) applying econometric cross-sectional and time series models to explain stock returns [19, 23], 2) constructing portfolios [22, 31, 43, 44], 3) forecasting stock returns [13], and 4) measuring comovement between investor sentiment and stock returns [2].

Some popular metrics of investor attention and sentiment, based on the content of various sources of information transmission, are given in Table 1.

**Table 1:** Summary of attention and sentiment metrics from the review of previous research.

Attention and sentiment metrics	Source article
Information from the issuer	
The tone of a company’s financial statements and conference calls	[45]
The tone of press releases and interviews with top managers	[8, 46]
Alphabetism and naming	[47, 48]
Information from analysts and professional investors	
Target prices	[49, 50]
Analysts’ recommendations (buy / hold / sell)	[42]
The degree of consensus in analysts’ forecasts	[49]
News and headlines	[4, 25, 33, 38, 40]
Information from stock exchanges, the mass-media and social networks	
Trading volume	[4, 15, 28, 31]
Extreme past returns	[4, 15]
Volatility	[32, 51]
Intensity of web search queries for companies (in Google, Wikipedia, etc.)	[15, 16]
Survey sentiment indicators	[14]
The tone of messages in social networks	[14, 36, 41]
Divergence of investor opinion	[13]
Author-developed indices based on the tone of investor messages and divergence of opinion (Bull-Bear Spread, Bullishness Index, Agreement Index, Variation of Bullish Ratio, etc.)	[13-15, 23, 34]

Source: the authors’ classification

Note: Table 1 displays attention and sentiment metrics categorized by the source from which a retail investor obtains trading information to make a decision.

**Fig 1:**
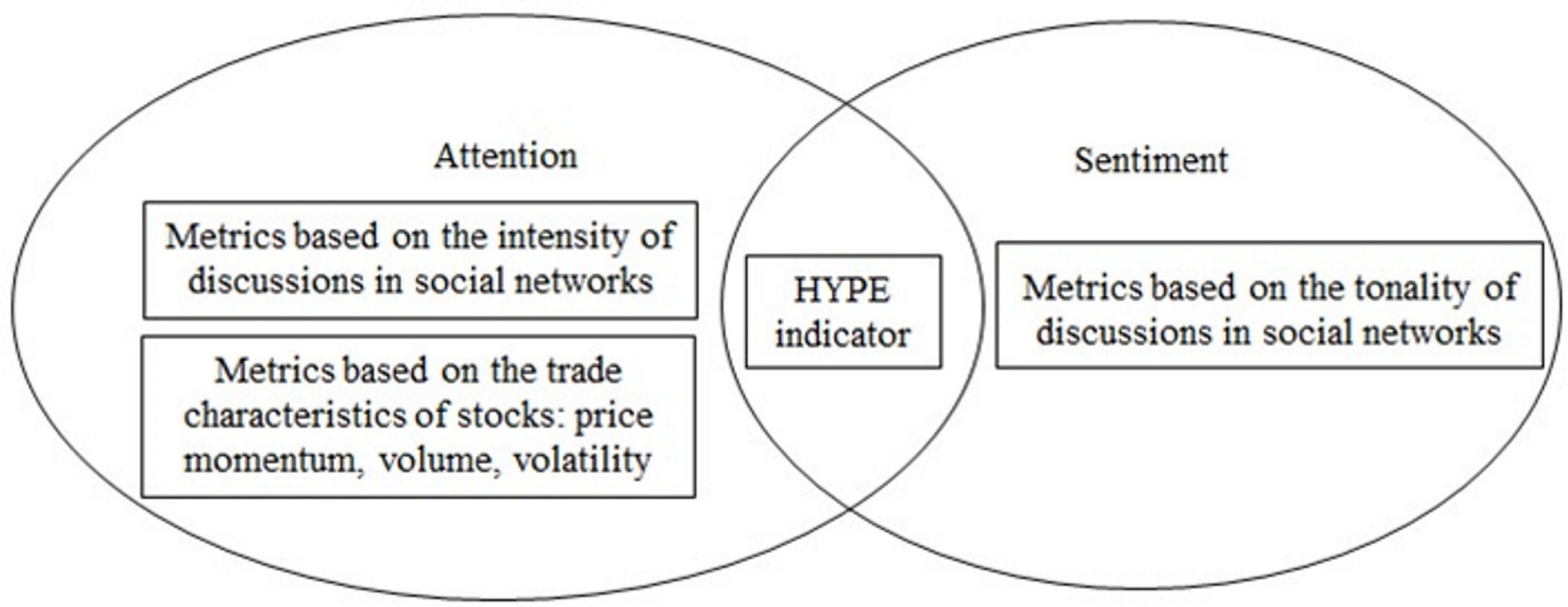
Metrics under consideration and the novel Hype indicator. Source: the authors’ classification.

The metrics considered in our study are presented in Fig 1.

We propose the following hypotheses:

H1. Social network communication is a more important characteristic in designing investment strategies than trading characteristics of assets.

We hypothesize that utilizing social capital (the views of the retail investment community on the investment attractiveness of a company) can yield higher returns than relying on stocks’ trading characteristics. Social activity and the opinion of ‘fellow’ investors are important for retail trading. Different metrics have different ‘memory’. We propose a novel Hype indicator that combines both the investor attention metric and the investor sentiment metric.

[52] showed that based on the analysis of investor sentiment in the StockTwits network using deep neural networks, it is possible to build an effective investment portfolio that beats benchmarks built without taking sentiment into account. In contrast to [52], where portfolios of a small number of stocks were considered and the weights changed, we consider portfolios of a larger number of stocks, the composition of which changes over time. [53] highlighted the significance of the influence of individual investor sentiment on the stock index returns in Germany, but the author used consumer sentiment index to measure the sentiment, while we consider investor sentiment based on messages in social networks.

[54] showed that it can be expected that stock markets in collectivist countries (as Russia) are more influenced by investor sentiment, while stock markets in individualistic countries, where people tend to give more weight to their own information and opinion, are more independent of the actions of the crowd.

[55] explored the European stock market for the link between stock prices and investor sentiment in Twitter and concluded that negative statements significantly affect prices. [56] reported that both negative and positive sentiment indices (based on the analysis of investment-related Twitter posts) are positively associated with abnormal returns, although the effect of positive sentiment is greater (negative sentiment was found to be insignificant for some periods). Our Hype indicator accounts for both the proportion of positive messages and the growth of messages.

H2a. Investment strategies taking into account sentiment characteristics are more profitable than investment strategies based on only attention characteristics.

The essence of the theory of attention is that there is an imbalance between supply and demand for a security (a stock). Individual investors tend to buy stocks that attract their attention, since they do not have enough time and resources to thoroughly study many stocks. But there is an asymmetry, because such a problem of analysis is not observed in the situation with the sale of stocks, because investors already own these stocks and have enough information about them. [57] constructed a mathematical model according to which stock returns and trading volume (as a metric of attention) are positively correlated. [27] found that the price of stocks with high trading volume during the day or week tend to grow. The higher popularity of a stock, driven by higher trading volume, increases the number of potential buyers but not the number of potential sellers, resulting in higher returns. [58] showed that the metrics of attention based on trade characteristics of stocks are significant for forecasting stock returns.

An alternative attention theory was proposed by [59]. Investors prefer to buy stocks that attract their attention more, which can be proxied by abnormal trading volumes, extreme returns, or a big news background. The stocks of companies that showed their ads during SuperBowl broadcasts from 1969 to 2001 demonstrated significant positive returns [60].

[61] compared different sentiment metrics based on text analysis using AI to study the influence of the sentiment of the East Money online platform on the daily stock performance in the Chinese stock market. The results showed that the positive sentiment of private investors generates positive stock returns the next day, and taking sentiment into account improves the quality of the forecasting model.

H2b. The both effects of sentiment- and attention-driven trading are more pronounced for low liquid stocks of small companies than for high liquid stocks of big companies.

Retail investors, whose actions are emotionally charged, constitute a large share of the trading volume of small-cap stocks. In contrast, for large companies, trading volume is mostly generated by institutional investors, who are less likely to act on emotion.

[22] argued that stocks of small companies are sensitive to sentiment to a greater extent. The authors’ explanation is that new information about small-cap stocks is rather scarce; any arrival of news encourages investors to commit trades, which results in abnormal returns. Small stocks are harder to arbitrage and harder to value than large stocks with a long and stable earnings history. Similar hypotheses about the effect of company size on sentiment in stock returns are discussed in [62, 63].

[15] found that the attention characteristic has the potential to contribute to abnormal returns for small and fast-growing companies rather than for large companies. [64] also concluded that investor attention has a greater impact on the idiosyncratic risk of small and fast-growing companies.

H2c. On a short time horizon, the characteristics of sentiment and attention allow an investor to build more profitable strategies. On a “long” time horizon (up to 6 months), the effects of sentiment and attention are smoothed out.

[39] concluded that very short investment intervals (intraday timeframes in minutes) do not ensure the profitability of investment strategies. [58] considered daily, weekly and monthly periods and found that the higher the frequency of measurement of stock sentiment, the higher its predictive power for stock returns. [65] considered daily data and revealed a negative reaction of stock returns for the current and future days depending on the sentiment of the current day.

[35] revealed a relationship between the average monthly return of country indices for 18 markets and past consumer sentiment in different time horizons (from 1 to 24 months). Investor sentiment significantly explains future negative returns on the time horizon of 1 month, while the impact on the time horizon of 6 months is insignificant. [35] used a proxy sentiment based on surveys of consumer behavior, while we do a textual analysis.

H3. A naive emotional strategy of copying the crowd’s behaviour (consensus trading) results in losses to investors.

We consider the crowd’s consensus one of the tools of spotting ‘meme stocks’ [2, 3, 23]. As a manifestation of crowd mentality and weak critical thinking, irrational behaviour of blindly following the crowd tends to result in losses to investors. We also hypothesize that the highest degree of consensus is observed for low liquid stocks of small and medium-sized companies, which are rarely spotted by the media and have a larger share of retail investors in their stocks’ trading volumes.

The authors of some previous studies argued that stocks for which a greater divergence of investor opinion is observed become riskier, thereby leading to potentially higher future returns. [66] found a positive significant impact of the divergence of investor opinion on stock returns in the US market. For the US market, similar results were obtained by [67]. [23] reported that in bear markets, a strong disagreement among investors is associated with higher returns. [68] concluded that overconfidence of most investors in a company may lead to a decrease in its future stock returns.

[59] showed that the trading of individual investors is highly correlated, giving rise to a systemic effect on pricing by this group of investors. The authors concluded that there is a herding of investment by retail (private) investors, and it is necessary to take into account the behaviour of individual investors who trade in a coordinated manner. [10] proved that the correlated sentiment of noise traders can change the equilibrium price of a stock in the market. [54] concluded that herding behaviour occurs during periods of downtrend in the Korean stock markets, but this behaviour was measured by a trade characteristic (volatility). [65] showed for the Australian market that the higher the disagreement among investors, the higher the stock return on the current day and the two following days, i.e. investing "with the crowd" does not bring profit to an investor.

We overcome the shortcomings of earlier research by using a unique source dataset and up-to-date methods of textual analysis and taking advantage of an increase in the participation of retail investors in the Russian stock market and in social media networks (especially during the quarantine and self-isolation periods during the COVID-19 pandemic).

## The Russian market survey, stock sample and social networks description

The Russian stock market began to function in 1992. Its feature is the tendency of investors to collectivism, which is due to the state policy of the former USSR, in which individualism was eliminated. The Russian stock market boomed from 2002 to mid-2008, due to rising oil prices: many companies entered the market, capitalization grew from 33% GDP in 2002 to 115% GDP, liquidity was high (source: NAUFOR (http://naufor.ru/getfile.asp?id=5338. In Rus.)). Beginning in mid-2008, problems arose due to the global financial crisis, in the period of relative stability in 2011–2013, the capitalization grew from 33.6% to 38.3% GDP (source: World Bank database). In 2014, because of the geopolitical crisis (the conflict with Ukraine) and falling oil prices, the market capitalization decreased to 18.7% GDP (source: World Bank database). In 2016–2017, there was a market recovery, but after that rapid growth was not observed. In 2019 and 2020, the market capitalization was 46.7% and 46.6% GDP, respectively (source: World Bank database). We note that since 2018, due to reducing inflation and lower yields on bank deposits, the influx of individuals to the stock market has increased. This inflow increased even more during the COVID-19 pandemic amid the provision of various benefits, and in 2020 the share of individuals in stock trading was more than 60% (source: MOEX).

The descriptive statistics on the Russian market and a number of other emerging markets for 2019–2021 is shown in S1 Appendix (Table A.1 in S1 Appendix). The Russian market is characterized by an extremely low P/E (5.8 in 2021, 10.9 in 2020) and not high market capitalization (841.9 USD bln in 2021, 694.7 USD bln in 2020) and trading volume (407.3 USD bln in 2021, 276.6 USD bln in 2020). Annual inflation in Russia was about 3.4–4.5% in 2019–2020, but it reached 8.4% in 2022, which was higher than in China, India and Brazil.

The sample consists of stocks of 60 companies, while the stock index contains securities of 43 companies. The trading data spans from January 1, 2014 to April 1, 2020. The data include stock splits and mergers and exclude dividends. The sample is divided into three equally sized groups:

1. Blue chips. These are constituents of the MOEX Russia Index and are cross-listed on the LSE, NYSE, or Frankfurt Stock Exchange. The average daily trading volume exceeds RUB100 million ($1.62 million as of October 2022). All the stocks are also or were constituents of the MOEX10 Index.
2. Stocks of medium-sized companies. The average daily trading volume ranges between RUB10 million and RUB100 million. All the stocks are constituents of MOEX sectoral indices and have been constituents of the MOEX Russia Index at least once.
3. Low liquid stocks of small companies that are not constituents of the MOEX Russia Index and are not cross-listed abroad.

We analyse the discussions of retail investors in social media networks. To do so, we examined two independent, open access online platforms that store message history. Telegram, with its variety of investment-related chats, is a relatively new channel that has been growing in popularity. The other, relatively older forum is the MFD forum, which is the most popular online platform for discussing investment-related topics in Russia.

The total number of active brokerage accounts in Russia amounts to 596,607 (As of April 2020, the Moscow Exchange defines active accounts as accounts with at least one security transaction per calendar month (https://www.moex.com/s719)). When combined together, the total number of users of the MFD forum (60,000) and of the Telegram channels that we monitored (975,839, with the number of unique users being close to 200,000) amounts to 43% of active accounts on the Moscow Exchange as of 2020. Compared to the US market, retail investors prevail in Russia (partly due to a rather poor development of collective investment schemes, such as mutual funds and non-government pension plans). Since 2014, the number of active accounts of retail investors has grown more than sevenfold.

The audience of the MFD forum and Telegram investment-related channels is comparable to the audience of conventional media (newspapers, magazines, television). In May 2020, active users of the MFD forum totalled 59,000 people, while subscribers to the most popular finance-related Telegram channel totalled 108,000 people (For comparison, the number of subscribers to Russia’s leading economic newspaper ‘Vedomosti’ amounted to about 200,000 people as of October, 2019 as stated by Mediascope).

One specific feature of the MFD forum is that the subject of discussion on every topic is a particular company, so there is no need to check what company the message refers to. In contrast, Telegram channels and chats are not dedicated to specific companies.

We suppose that the attention characteristic will not act the same for blue chips and for third-tier stocks. To account for this, we composed equally weighted portfolios of stocks with large, mid, and small market capitalizations.

One advantage of sentiment analysis in the Russian stock market is that Russian stocks are rarely discussed in languages other than Russian. This is especially true for small and mid-sized companies whose stocks are not cross-listed abroad. In contrast, American stocks from the S&P 500 Index are a subject of discussion by retail investors in many languages both within and outside the US. For instance, the trading volume of US stocks on the SPB Exchange alone amounted to $450 million in April 2020 (https://fomag.ru/news/roman-goryunov-potentsial-uvelicheniya-kolichestva-torguemykh-instrumentov-eshche-ne-ischerpan/). Because of the popularity of US stocks worldwide, it is impossible to thoroughly and comprehensively analyse investor sentiment of US stocks, which would result in less reliable conclusions.

The descriptive statistics on our sample is given in Table 2. The Russian stock market is highly volatile even in periods of macroeconomic stability (2017–2019). The whole period under consideration includes financial crises and high turbulence intervals: 2014–2015 and 2020.

**Table 2:** The descriptive statistics on our sample.

Indicator	Low liquid stocks of small companies	Stocks of medium-sized companies	Blue chips	All stocks in our sample
Number of instruments	20	20	20	60
Market capitalization:				
• USD bln	0,25	3,47	22,14	8,36
• RUB bln	14,91	202,38	1 307,44	493,08
Average monthly return:				
• 2014–2020	2,53%	1,00%	1,26%	1,60%
• 2017–2019	0,69%	0,14%	0,73%	0,52%
Average monthly volatility,				
• 2014–2020	22,26%	10,37%	8,96%	13,86%
• 2017–2019	16,71%	8,17%	6,97%	10,61%
Average monthly trading volume, RUB bln	0,00244	0,11037	1,58710	0,56664
Momentum premium 1-0-1 (average monthly alpha)	0,58%	0,31%	0,56%	0,56%
Average monthly share of negative messages	15,18%	18,38%	23,26%	18,94%
Average monthly share of positive messages	15,26%	17,12%	17,19%	16,52%
Average monthly number of all messages	457,14	777,70	2 129,84	1 121,56
Average monthly value of the HYPE indicator	10,699	10,579	6,468	9,248

Source: the authors’ calculations

## Methodology

### Authors’ modification to neural networks, textual processing, and assessment algorithms

To build a sentiment index for every company in the sample, we had to solve the problem of processing a large array of messages (statements) retrieved from finance-related social networks designed for Russian market participants. It should be noted that users of social media platforms express their thoughts in writing in many ways such as modifying company names, replacing words with jargon (e.g., using the CEO’s name or the beneficiary owner’s name, the name of the parent company) therefore, making it impossible for us to proceed without special text processing techniques. Unlike in the case of newspaper and magazine articles or official communications, we cannot label messages as negative if they contain the word ‘down’. This is partly due to peculiarities of the Russian language, which offers a wide variety of ways of expressing emotion, and partly due to a relatively young age of the users.

At the stage of data preprocessing, we performed lemmatization and stemming to convert words into their root form, removed endings, stop words, and punctuation marks. As a result, the distribution of the number of word repetitions became more uniform, and the share of outliers decreased. Then, we compiled a special Russian-language dictionary that contained official and informal companies’ names as well as their respective ticker symbols.

The next step in building basic machine learning models is to convert messages to numeric vectors. For this, we applied One Hot encoding and TF-IDF encoding, which allowed us to reduce the significance of words that had no practical meaning.

A neural network was used to assign the probability for a message to belong to a certain sentiment class. We distinguished three classes of investor sentiment: 1) negative (i.e., messages are associated with a bearish trend and a sell recommendation), 2) positive (i.e., messages contain forecasts of price growth and recommendations, often disguised, to take a «long» position), and 3) neutral (i.e., messages do not provide a clear clue as to how the participants in the discussion will act).

To solve the classification problem via supervised learning, we first applied the model of [61], which is a neural network that finds patterns in words in messages given information on their *a priori* semantic relationship.

To assess the superiority of the neural network (the model of [61]), we used standard machine learning techniques for our array of messages as a benchmark. The purpose of the comparison is to assess the degree to which the collected and manually labelled data are informative. The results are given in Table 3.

**Table 3:** Results of textual processing using a neural network and machine learning techniques.

Approach	One-Hot encoding						TF-IDF					
	Acc (Train)	Acc (Test)	F_1_ (Train)	F_1_ (Test)	F1 weigh (train)	F1 weigh (test)	Acc (Train)	Acc (Test)	F_1_ (Train)	F_1_ (Test)	F1 weigh (train)	F1 weigh (test)
Stochastic gradient descent	0.812	0.728	0.710	0.564	0.811	0.732	0.769	0.729	0.625	0.563	0.775	0.734
Random forest	0.984	0.736	0.977	0.524	0.984	0.756	0.989	0.744	0.983	0.507	0.989	0.782
Decision tree	0.785	0.550	0.742	0.455	0.771	0.511	0.890	0.614	0.856	0.472	0.885	0.591
Constant model	0.751	0.609	0.286	0.252	0.644	0.461						

Source: the authors’ calculations

Note: Table 3 contains the results of model tests on the training and test samples for two types of metrics: Accuracy and F1-score. Accuracy shows the percentage of correctly classified responses. The macro-averaged F1-score takes into account the qualitative features of the dataset that affect the learning process, including the problem of class imbalance. Therefore, we select the best model according to the F1-score. The best model for the test sample is the Stochastic gradient descent: its F1-score is 0.564 for one-hot encoding and 0.563 for TF-IDF.

Next, we applied the CNN-transformer model proposed in [69] for data analysis. Since One-Hot encoding can lead to overfitting and fails to properly handle informal, frequently changing vocabulary, we used the CNN embedding procedure (https://tfhub.dev/google/universal-sentence-encoder-multilingual-large/3) in a definitive version of the model.

After CNN embedding, we used a fully connected neural network with three layers. The first layer has 128 neurons and the second has 64 neurons. In both layers, the hyperbolic tangent function was used as the activation function. We also added dropout regularization with p = 0.5 after each layer to avoid overfitting problems. The last layer consisted of three neurons and has a softmax activation function. The model was trained for 20 epochs with a learning rate of 0.0015. The CNN-based model produced an F1-score of 57.7% and an Accuracy of 69.4% on the test sample.

The collection and analysis method complied with the terms and conditions for the source of the data.

### Attention and sentiment metrics and portfolio designs

After the array of messages was assorted across the three classes, we constructed metrics of positive and negative investor sentiment (Table 4).

**Table 4:** Key filters for portfolio designs.

Sentiment / attention proxy	Name of a variable	Definition
The novel integral indicator of investor sentiment and attention		
Hype Indicator	*Hype*	The indicator accounts both for an increase in the number of positive messages and a growth of the total number of messages. Its value ranges from 0 (all messages are neutral or negative or the total number of messages has decreased / has not changed) to 10 (all messages are positive and the total number of messages has increased 10 times or more)
Indicators of investor sentiment derived from social network analysis		
Growth of positive messages	*positive*	A relative change in the number of positive messages for each company
Growth of negative messages	*negative*	A relative change in the number of negative messages for each company
Consensus	*consensus*	This indicator assesses the consensus of investor opinion. Its value ranges from 0 (50% of negative and 50% of positive messages) to 1 (100% of positive or 100% of negative messages)
Indicators of investor attention derived from social network analysis		
Growth of the total number of messages	*messagesum*	A relative change in the total number of messages about a company posted in the MFD forum and on Telegram channels
Relative attention	*relative_attention*	A share of messages about a company relative to the total number of messages across all companies for a period. The indicator reveals the most discussed stocks in a portfolio (which are mostly blue chips)
Indicators of investor attention derived from trading characteristics		
Price momentum	*momentum*	The profitability of a market-neutral portfolio of stocks with the best and worst performance. It is calculated as the return on the portfolio of 50% of stocks with the highest return minus the return on the portfolio of 50% of stocks with the lowest return over the previous month
Volatility	*volatility*	The profitability of a market-neutral portfolio of stocks with the largest and smallest volatility growth. It is calculated as the return on the portfolio of 50% of stocks with the highest average daily volatility minus the return on the portfolio of 50% of stocks with the lowest average daily volatility over the previous month
Trading volume	*volume*	The profitability of a market-neutral portfolio of stocks with the largest and smallest trading volume (in money terms). It is calculated as the return on the portfolio of 50% of stocks with the largest increase in trading volume minus the return on the portfolio of 50% of stocks with the lowest increase in trading volume over the previous month

Source: the authors’ selection

Note: We use different groups of metrics to design portfolios: indicators of investor attention and sentiment based on social network analysis, our Hype indicator that integrates metrics of investor attention and sentiment, and traditional indicators of attention based on trading characteristics.

Then we introduced the new Hype indicator, which integrates the metrics of investor attention and sentiment. It equally accounts for the proportion of positive messages and for increased intensity of discussion with respect to a particular stock. The indicator is calculated as follows:

$$Hype_{T}=\frac{MSG_{T}^{POSITIVE}}{MSG_{T}^{POSITIVE}+MSG_{T}^{NEGATIVE}}\cdot (\frac{MSG_{T}^{ALL}}{MSG_{T-1}^{ALL}}-1)$$ (1)

where ${MSG}_{T}^{POSITIVE}$ is the number of positive messages for each company, ${MSG}_{T}^{NEGATIVE}$ is the number of negative messages for each company, ${MSG}_{T}^{ALL}$ is the sum of positive, negative, and neutral messages for each company, $\left(\frac{{MSG}_{T}^{ALL}}{{MSG}_{T-1}^{ALL}}-1\right)$ is a change in the total number of messages from the previous month. If the number is less than zero, then the variable assumes the value of zero.

If we composed a portfolio of stocks with the largest proportion of positive messages, then we are most likely to overlook the intensity of discussion. For instance, there could be merely 20 messages for a stock, all of which are positive. However, this does not imply that such a barely discussed security would attract much investor attention. On the other hand, if we composed a portfolio of stocks only based on the growth of messages, we may be left with stocks whose ratio of positive and negative messages is 50/50, and thus our conclusions could be wrong. Our Hype indicator overcomes these shortcomings by identifying stocks that are most actively and positively discussed. We chose positive rather than negative messages as short selling opportunities are unavailable for many stocks in the low liquid environment of the Russian market.

We used a number of indicators to assess the impact of investor attention and sentiment (Table 4).

To evaluate portfolio performance, we adopted the cross-sectional momentum strategy proposed in [70] without a 1-month time lag. Portfolio profitability is calculated as a weighted average so that all stocks in the portfolio are initially assigned equal weights. The portfolio of winners (losers) is composed of stocks that have the largest (lowest) values of the sentiment metric and which account for 50% of the total ordered sample of 60 stocks (indiscriminate with respect to issuers’ market capitalization), and of ordered subsamples of ten stocks (grouped based on issuers’ market capitalization). The number of securities in the portfolios is constant throughout the testing period.

We examined the performance of these portfolios over three different time periods:

1. In the short run with a 1-month formation period and a 1-month holding period;
2. In the medium run (relatively) with a 3-month formation period and a 3-month holding period;
3. In the «long» run (relatively) with a 6-month formation period and a 6-month holding period.

The choice of such time periods is due to the particularities of the Russian emerging market and a relatively recent history of messages on Telegram channels. We composed an equally weighted portfolio of 60 stocks from the initial sample to detect irrational behaviour among investors. For each subsample, a corresponding equally weighted benchmark was also composed. For the details of the methodology see S2 Appendix (Fig B.1 in S2 Appendix).

We formed portfolios by applying metrics of investor attention and sentiment (Table 4) and evaluated their performance with performance metrics such as average monthly return, excess return, and alpha (excess return associated with risk). The group of winner portfolios includes 50% of the sample stocks with the highest performance scores, and the group of loser portfolios includes 50% of the sample stocks with the lowest ones.

Winners minus losers (WMLs) are a market-neutral portfolio with a «long» position in winners and a short position in losers. Although the WML portfolio may reveal the impact of investor attention and sentiment, we had to consider the portfolios of winners and losers separately because taking short positions in the Russian stock market is only feasible for very liquid stocks. Additionally, it is inappropriate to compare the performance of a market-neutral portfolio against an equally weighted benchmark.

## Empirical evidence of attention and emotional trading

To find evidence of attention and emotional trading in the Russian stock market, we applied the [70] strategy to nine portfolios designed on the basis of attention and sentiment metrics over three distinct time periods (1, 3, and 6 months). The results of empirical tests of the presence of attention and emotional trading for different portfolio designs for a short period of 1 month and of 6 months for the overall sample of 60 stocks are given in Tables 5 and 6, respectively. Complete and more detailed results for the overall sample and for the three subsamples are given in S3 Appendix (Tables C.1, C.2 in S3 Appendix) (The full results are presented in the authors’ site: http://fmlab.hse.ru/appendices).

**Table 5:** The performance of the nine portfolios over a short period (1 month).

	Alpha	Mean monthly excess return	Mean monthly return
1. Winners			
1.1 The integral Hype indicator			
Hype	0.39 (1.5)	0.58** (2.23)	2.17*** (3.03)
1.2 Indicators of investor sentiment based on social network analysis			
positive	0.47 (1.5)	0.42 (1.4)	2.01*** (3.08)
negative	0.55* (1.89)	0.66** (2.38)	2.25*** (3.22)
consensus	0.17 (0.73)	0.2 (0.91)	1.8*** (2.75)
1.3 Indicators of investor attention based on social network analysis			
messagesum	0.64** (2.27)	0.57** (2.12)	2.17*** (3.41)
relative_attention	0.53* (1.87)	0.29 (1.03)	1.89*** (3.26)
1.4 Indicators of investor attention based on trading characteristics			
price momentum	0.56* (1.95)	0.65** (2.39)	2.25*** (3.24)
volatility	0.4 (1.31)	0.48 (1.63)	2.07*** (2.99)
volume	0.36 (1.2)	0.23 (0.82)	1.83*** (2.93)
2. Losers			
2.1 The integral Hype indicator			
Hype	-0.19 (-0.66)	-0.37 (-1.34)	1.23** (2.06)
2.2 Indicators of investor sentiment based on social network analysis			
positive	-0.44 (-1.55)	-0.58** (-2.12)	1.02 (1.66)
negative	-0.35 (-1.22)	-0.46 (-1.65)	1.14* (1.82)
consensus	-0.15 (-0.62)	-0.21 (-0.9)	1.39** (2.21)
2.3 Indicators of investor attention based on social network analysis			
messagesum	-0.5* (-1.95)	-0.73*** (-2.84)	0.87 (1.52)
relative_attention	-0.4 (-1.49)	-0.44* (-1.74)	1.16* (1.81)
2.4 Indicators of investor attention based on trading characteristics			
price momentum	-0.49* (-1.71)	-0.57** (-2.08)	1.03 (1.62)
volatility	-0.25 (-0.9)	-0.36 (-1.34)	1.23* (1.98)
volume	-0.35 (-1.18)	-0.23 (-0.8)	1.37* (1.93)
3. WML			
3.1 The integral Hype indicator			
Hype	0.58 (1.09)	-0.65 (-0.95)	0.95* (1.81)
3.2 Indicators of investor sentiment based on social network analysis			
positive	0.91* (1.7)	-0.6 (-0.79)	1.0* (1.97)
negative	0.9 (1.59)	-0.48 (-0.65)	1.11** (2.05)
consensus	0.32 (0.68)	-1.19 (-1.64)	0.41 (0.91)
3.3 Indicators of investor attention based on social network analysis			
messagesum	1.14** (2.44)	-0.3 (-0.43)	1.3*** (2.91)
relative_attention	0.93* (1.93)	-0.86 (-1.06)	0.73 (1.59)
3.4 Indicators of investor attention based on trading characteristics			
price momentum	1.05* (1.84)	-0.38 (-0.5)	1.22** (2.25)
volatility	0.66 (1.14)	-0.76 (-0.99)	0.84 (1.52)
volume	0.71 (1.19)	-1.13 (-1.27)	0.46 (0.81)
Here and below: ***—the significance at the 1% level, **—at the 5% level, *—at the 10% level			

Source: the authors’ calculations

Note: By ‘excess return’, we mean the monthly return of the constructed portfolio (based on a chosen characteristic) minus the monthly return of the benchmark. By the benchmark, we mean a portfolio of stocks of companies in our sample with equal weights: either all 60 companies, or 20 companies that form one or another group by capitalization. By ‘alpha’, we mean the risk-adjusted excess return based on regression constructions (regression factors are shown in Table 5 in the first column).

Winner portfolios composed based on the total number of messages, relative attention to a company, negative sentiment, and price momentum have positive and significant alphas. The following winner portfolios generate significant and positive mean monthly excess returns: composed based on the Hype indicator, negative sentiment, the total number of messages, and relative attention to a company. The Hype winner and loser portfolios offer an investor a mean monthly return of 2.17% (statistically significant at 1%) and 1.23% (statistically significant at 5%), respectively.

Alphas of all loser portfolios are negative. Also, portfolios composed based on the total number of messages and price momentum have significant negative alphas and mean monthly excess returns.

The following WML portfolios have positive and significant alphas: composed based on positive sentiment, the total number of messages, relative attention to a company, and price momentum.

**Table 6:** The performance of the nine portfolios over a «long» period (6 months).

	Alpha	Mean monthly excess return	Mean monthly return
1. Winners			
1.1 The integral Hype indicator			
Hype	0.383 (0.97)	0.18 (0.61)	2.217*** (3.12)
1.2 Indicators of investor sentiment based on social network analysis			
positive	0.075 (0.14)	-0.6 (-1.33)	1.437** (2.34)
negative	-0.005 (-0.01)	-0.638 (-1.26)	1.397* (2.08)
consensus	-0.265 (-0.68)	-1.008** (-2.61)	1.027* (1.91)
1.3 Indicators of investor attention based on social network analysis			
messagesum	0.607 (0.89)	-0.053 (-0.1)	1.983** (2.86)
relative_attention	0.518 (1.35)	0.428 (1.53)	2.463*** (3.32)
1.4 Indicators of investor attention based on trading characteristics			
price momentum	0.673 (1.27)	-0.277 (-0.54)	1.758*** (3.23)
volatility	0.615 (1.29)	-0.243 (-0.53)	1.792*** (3.31)
volume	0.2 (0.38)	-0.015 (-0.04)	2.02** (2.69)
2. Losers			
2.1 The integral Hype indicator			
Hype	-0.23 (-0.62)	-0.108 (-0.4)	1.927** (2.38)
2.2 Indicators of investor sentiment based on social network analysis			
positive	-0.24 (-0.41)	0.342 (0.72)	2.377** (2.33)
negative	-0.1 (-0.24)	-0.155 (-0.5)	1.88** (2.46)
consensus	0.123 (0.32)	1.223** (2.53)	3.26** (2.84)
2.3 Indicators of investor attention based on social network analysis			
messagesum	-0.073 (-0.15)	-0.603 (-1.48)	1.432** (2.22)
relative_attention	-0.085 (-0.23)	-0.75* (-2.12)	1.287** (2.33)
2.4 Indicators of investor attention based on trading characteristics			
price momentum	-0.705 (-1.34)	0.262 (0.51)	2.297* (2.03)
volatility	-0.648 (-1.38)	0.227 (0.49)	2.263* (2.08)
volume	-0.2 (-0.38)	0.015 (0.04)	2.05** (2.32)
3. WML			
3.1 The integral Hype indicator			
Hype	0.613 (0.84)	-1.747 (-1.76)	0.29 (0.53)
3.2 Indicators of investor sentiment based on social network analysis			
positive	0.317 (0.29)	-2.977* (-2.12)	-0.94 (-1.05)
negative	0.095 (0.1)	-2.518* (-2.2)	-0.483 (-0.68)
consensus	-0.388 (-0.6)	-4.267** (-2.94)	-2.232** (-2.77)
3.3 Indicators of investor attention based on social network analysis			
messagesum	0.682 (0.8)	-1.485 (-1.51)	0.552 (0.89)
relative_attention	0.602 (1.06)	-0.858 (-1.3)	1.178** (2.56)
3.4 Indicators of investor attention based on trading characteristics			
price momentum	1.378 (1.3)	-2.573 (-1.61)	-0.538 (-0.53)
volatility	1.263 (1.33)	-2.507 (-1.67)	-0.472 (-0.51)
volume	0.398 (0.38)	-2.067 (-1.77)	-0.03 (-0.04)

Source: the authors’ calculations

Note. No portfolios are characterized by a significant alpha. The winner portfolio based on our Hype indicator allows an investor to obtain a positive alpha and excess return and a positive and significant monthly return. Winner portfolios based on the price momentum, volatility and the number of all messages are also characterized by positive and relatively high alphas.

Alphas of all loser portfolios are negative, except for the consensus metrics. The loser portfolio based on the consensus metrics demonstrates positive and significant excess return.

A WML portfolio based on the activity of discussions (relative_attention) has a positive alpha and a positive and significant monthly return.

As seen from Table 5, the winner portfolio of stocks with the highest values of the Hype indicator produced a positive and significant return in excess of the benchmark in the short run (1 month). Alpha for this portfolio was positive but not significant. The loser portfolio with the lowest values of the Hype indicator showed a lower mean return, and its excess return was negative. Thus, in the short run, the attention characteristic is of practical interest in the Russian market and can outperform the market (without risk adjustment). In the «long» term (6 months), the winner portfolio based on our Hype indicator allows an investor to obtain a positive alpha and excess return and a positive and significant monthly return (Table 6).

In the short run, the winner portfolio of the most intensely discussed stocks (which exhibit the largest increase in the total number of messages over the previous month) outperformed all other winner portfolios based on the metrics of investor sentiment and attention (Table 5), the alpha was significant and positive. The excess return of the winner portfolio composed based on the growth of negative messages was higher than that of the Hype portfolio, and the alpha was significant and positive. We explain this by overreaction: negative discussions in the previous month cause a significant decrease in stock prices, and next-month investors have an opportunity to buy undervalued stocks. There is no similar observation for the winner portfolio composed based on an increase in the number of positive messages: if investors are extremely positive about a company, then its abnormal return is positive, but its statistical significance is rather weak. This is in line with the results in [57] that both negative and positive sentiment indices (derived from the analysis of Twitter posts) are positively associated with abnormal returns. The authors also found that the role of positive sentiment is greater. However, [57] did not investigate the effects of positive and negative sentiment over different time periods, while we considered three distinct periods.

Over a relatively «long» period of 6 months (Table 6), investments in the most intensely discussed stocks are the best in terms of performance when the winner and WML portfolios were considered. They demonstrated high, positive and significant mean monthly returns and positive alphas (but the statistical significance of alphas is not high). We explain this by the fact that blue chips receive the most attention, while investors pay less attention to less liquid stocks of small and medium-sized companies. Thus, in the «long» run, liquid stocks of large companies can be chosen to construct a profitable strategy. Our results regarding the impact of the number of messages on social media platforms and the discussion activity on stock returns in the emerging Russian market are in line with findings on the impact of investor attention in developed markets in [27], where investor attention was measured by trading volume. At the same time, our results are not consistent with the conclusions of [30] on the emerging stock market in Indonesia.

The winner portfolio composed based on investor consensus opinion produced insignificant excess returns in the short run (Table 5). In the «long» run, on the contrary, the winner portfolio based on consensus of investor opinions, produced significant but negative excess return and negative alpha (Table 6). So, following the crowd is not profitable.

In the short term, for portfolios composed based on attention metrics derived from trading characteristics, the momentum portfolio allowed an investor to obtain positive and significant alpha and excess returns (Table 5). So, an investor can outperform the market. In the «long» term, the alphas of portfolios based on trading characteristics were positive, but insignificant (Table 6).

Over a short period, all loser portfolios had negative alphas and excess returns (Table 5). We offer the following explanation: these stocks ‘abide’ in an information vacuum. If stocks do not attract investor attention, then investment in such stocks may not be profitable. The same is true for the «long» run, except for the consensus metrics: the loser portfolio based on the consensus metrics demonstrates positive and significant excess return. So, it is possible to outperform the market (not taking into account risk adjustment).

We explain negative excess returns on WML portfolios by asymmetric returns: although some winner portfolios were superior to the loser portfolios in terms of excess returns, the returns on loser portfolios were also positive (Table 5). This means that investors cannot outperform the benchmark over a short period. Positive and significant alphas were produced by WML portfolios composed based on the total number of messages for a company and its presence in discussions (a manifestation of investor attention), the growth of positive messages, and price momentum.

**Fig 2:**
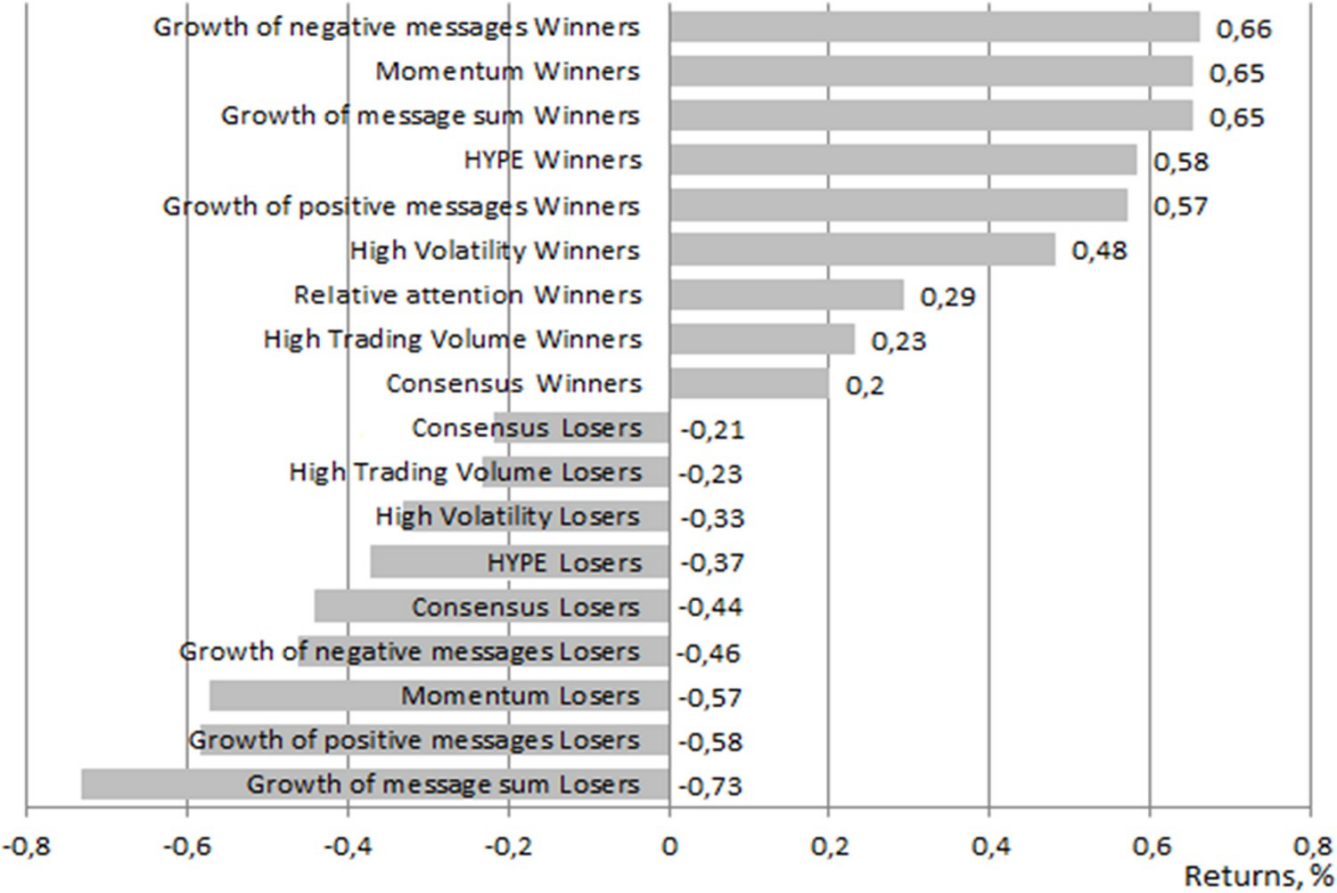
Monthly excess returns on each winner and loser portfolio over a short period. Source: the authors’ calculations. Note: Over a short period, the HYPE winner portfolio generated a monthly excess return of 0.58% to an investor. The winner portfolio composed based on the growth of negative messages generated a monthly excess return of 0.66% to an investor.

Portfolios ordered by monthly excess returns for each period are shown in Fig 2 and in Fig D.1 and D.2 of S4 Appendix. In the short run, all winner portfolios showed positive excess returns and superiority over losers (Fig 2), and some of winner portfolios showed positive and significant alphas (Table 5). We explain that it is due to the attention effect: stocks that do not attract positive or negative investor attention do not provide investors with an opportunity to obtain positive excess returns.

In the short run, the winner portfolio composed based on the growth of negative messages delivered the significant positive alpha and highest excess return, which is very close to the excess return on portfolios composed based on the largest growth of the total number of messages and momentum (Table 5). The loser portfolio of stocks with the smallest increase in the total number of messages exhibited the poorest performance (a negative significant alpha).

In the medium run, the portfolio ranking by excess returns changes (Fig D.1 in S4 Appendix). The portfolio with the lowest consensus of investor opinion achieved the highest and significant excess return. Picking stocks with the highest trading volume and the value of the Hype indicator allows an investor to obtain positive excess returns. In the «long» run, we also observed that the portfolio with the lowest consensus of investor opinion achieved the highest and significant excess return (Fig D.2 in S4 Appendix, Table 6). We explain this by the fact that the highest degree of consensus prevails for low liquid stocks of small and medium-sized companies, which are relatively rarely discussed.

All WML portfolios delivered positive returns and alphas in the short run (some of alphas are also significant) and negative returns in the medium run. Thus, it seems that behavioural biases are short-lived in nature and that their effect fades over time.

For the metrics of sentiment derived from social network analysis, such as the growth of positive and negative messages, and for the attention metric based on the growth of the total number of messages, as the holding period increases, the return tended to decrease almost linearly for the winner portfolios and increased linearly for the loser portfolios (Fig 2, Fig D.1, D.2 in S4 Appendix). A linear decrease in returns as the holding period increases was also observed for the WML portfolios. It should be noted that the loser portfolio composed of stocks that have the smallest number of positive messages delivered losses to investors over periods of 1 and 3 months but delivered a positive excess return over a period of 6 months (the statistical significance is not high). The excess return of the portfolios of stocks with the smallest increase in negative messages is negative for all three periods. We explain this by the fact that an excessively positive discussion tone does not adequately reflect current events and prevents investors from obtaining positive returns.

**Fig 3:**
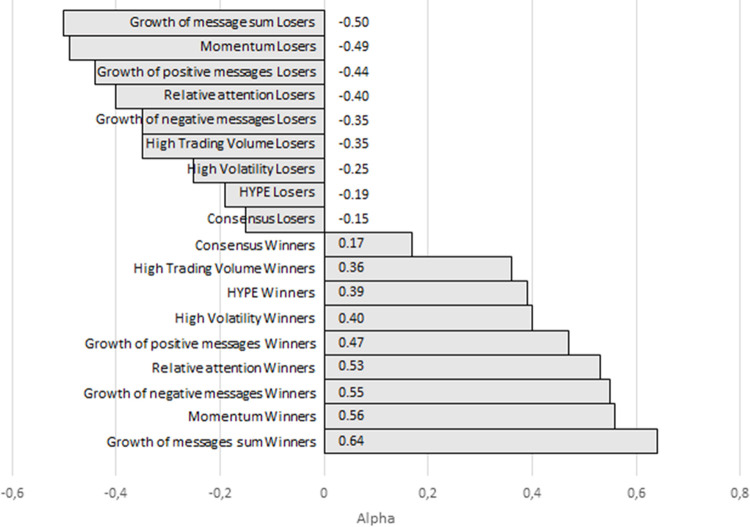
Alphas on each winner and loser portfolio over a short period. Source: the authors’ calculations. Note: Over a short period, the HYPE winner portfolio generated a positive alpha of 0.39. The winner portfolio composed based on the growth of messages generated the best alpha of 0.64.

In addition to portfolios composed based on sentiment and attention metrics derived from social network analysis, we composed portfolios based on stocks’ trading characteristics for comparison purposes (Figs 2 and 3, Fig D.1, D.2 in S4 Appendix). Investment in the winner portfolios formed by traditional factors (trading volume, volatility, and momentum) provided investors with a positive excess return in the short run (1 month). The excess return and the alpha for price momentum were significant. In the medium run, however, the role of traditional factors decreased, and in the «long» run, the excess return of such portfolios became negative (the alphas are positive, but insignificant). In contrast, the loser portfolios formed by traditional factors generated losses in the short run but tended to provide investors with positive excess returns in the medium and «long» run. The WML portfolios delivered positive excess returns in the short run and negative returns in the medium and «long» runs.

For the volatility and trading volume portfolios, increasing the holding period from 3 months to 6 months allowed investors to reduce their average losses. Our results are in line with those of [71] for the Korean stock market. The authors found that the momentum factor is associated with negative performance for all arbitrage portfolios. However, over a period of 12 months, loser portfolios tended to exhibit a reversal and instead brought positive returns. [71], based on the analysis of previous literature, also concluded that unlike in developed markets, momentum strategies are rarely profitable in Asian stock markets.

Our results are partly in line with the results in [31]. [31] revealed that speculative stocks in the Chinese market tend to be excessively overvalued over a short period followed by a period with a relatively low return. The authors argued that for the entire Chinese markets, stocks with both high and low trading volumes tend to underperform the market, and this effect is stronger for high trading volume portfolios over a period of up to 100 days. [28] reported that in the US and Korean markets, behavioural biases lead to a more profound reversal in prices of winner/loser stocks with high trading volume.

We point out one peculiar result: for the Hype winner and Hype WML portfolios and for the most intensely discussed stocks, a U-shaped return pattern was observed across all considered periods (Fig 2, Fig D.1, D.2 in S4 Appendix). The Hype winner portfolio exhibited the greatest consistency across all periods, and was the only portfolio that generated positive excess returns in the medium run, but the statistical significance of alpha was not high. Investments in intensely discussed stocks over a short period of 1 month and a «long» period of 6 months yielded a positive excess return, but over a period of 3 months, we observed the overreaction effect in which the mean return turns negative. According to [4], positive investor attention is associated with higher stock returns in the short run and with the reversal effect in the «long» run. We witnessed a similar reversal effect over a medium-length period of 3 months. For the Chinese market, [15] reported that investor attention indicated by abnormal levels of the Baidu search volume index positively affects stock returns in the current period, but the relationship is reversed in the subsequent period. These results on the reversal effect of investor attention are consistent with the findings of [72] for developed markets. Attention metrics in their research were derived from Google search volume data.

Among the winner and WML portfolios, the one with the greatest losses in periods of 3 and 6 months was the portfolio composed on the basis of consensus of investor opinion (Fig 2, Fig D.1, D.2 in S4 Appendix), alpha and excess return were negative. So, in the Russian market, the strategy ‘Go against the crowd’ can be profitable. Our results are not consistent with the conclusions of [26] in that high divergence-of-opinion stocks (i.e., stocks for which the lowest degree of consensus is observed) yield less returns to investors in future periods. The author explained this by short-sale constraints and by excessive buying when the activity of optimistic investors causes overpricing of stocks in the current period and a decrease in price in the subsequent period. Our results are in line with those of [67]. These authors also revealed a significant and positive impact of divergence of investor opinion on stock returns. Their explanation was that high divergence-of-opinion stocks are riskier, which leads to higher future returns. Similar results were obtained for the US stock market in [68]. Thus, our results are an argument in favour of the risk hypothesis.

Next, we compared monthly excess returns of the loser portfolios for different periods. Investing in a portfolio of high divergence-of opinion stocks led to a negative excess return over a period of 1 month and to a positive excess return over periods of 3 months and 6 months. As before, we explain this by the fact that high divergence-of-opinion stocks are mainly liquid stocks of large companies.

Picking stocks with the lowest intensity of discussion (as measured by the Hype indicator, the growth of the total number of messages or by relative attention) resulted in losses to investors in the short and «long» run (Tables 5 and 6). Positive excess returns were observed only in the medium run, which we attribute to the overreaction effect.

Overall, our results confirmed the findings of [13] that the sentiment of retail investors measured by conventional metrics derived from social networks affects stock returns. However, as a step further, we reinforced these findings with the novel Hype indicator. Our results imply that the Russian stock market is inefficient.

## Robustness check

For the robustness check, we first considered excess returns of the winner portfolios from different market capitalization groups. Over a short period, the attention effect was stronger for large-cap stocks (Fig E.1 in S5 Appendix), but over a longer period, it was stronger for low liquid stocks of small companies. The alpha of the Hype portfolio of large-cap stocks was statistically significant (S3 Appendix).

A high degree of consensus on investor opinion was associated with low excess returns. This effect was more pronounced in the medium and «long» run for stocks of small companies (Fig E.1 in S5 Appendix). We concluded that investment in low liquid stocks generates the highest losses when compared to investment in stocks of medium-sized and large companies.

In the short and medium run, the momentum factor performed better for low liquid stocks (Fig E.1 in S5 Appendix). The statistical significance of the 1-0-1 and 3-0-3 investment strategies is shown in S3 Appendix (Tables C.1 and C.2 in S3 Appendix).

Contrary to our results, over a short period, [22] found that stocks of small companies are more sensitive to sentiment because new information about stocks of small companies is scarce so any arrival of news encourages investors to commit trades, thereby generating abnormal returns. [15] also documented that attributes such as small size and fast growth are likely to reinforce the impact of investor attention on excess returns. The authors measured investor attention by the number of search queries for a company and suggested that company-specific search queries capture the activity of retail investors to a larger extent than the activity of institutional investors.

On the other hand, [57] found that abnormal returns of large-cap stocks are more sensitive to investor sentiment, while small-cap stocks are less affected because given a relatively low number of investor messages, a few positive or negative comments can boost the relationship between sentiment and stock returns. Over a short period, our results are in line with the findings of [57].

Second, we verified the results under new conditions of more concentrated portfolios. Instead of 50% of the sample, we picked stocks that are best and the worst performing in each metric within 40% and 25% of the sample (Table E.1 in S5 Appendix). The mainstream results for the 40% portfolios were similar to those obtained for the 50% portfolios, but the statistical significance of alphas and excess returns of most of these portfolios was lower. For the 25% portfolios, we did not obtain any special and pronounced results.

Thus, our results are robust for different portfolio designs across three groups of companies by market capitalization. In the short run, the results for the portfolios composed based on sentiment and attention of retail investors were similar to those for the momentum portfolio. If an investor buys stocks guided by the activity on social media, the outcome will be similar to the outcome of simply following the trend. However, over medium- and «long»-term horizons, we observed another pattern: stocks most positively discussed in the past exhibited the memory effect, which is not revealed by basic metrics derived from trading characteristics.

A result that is different from previous findings is that the Hype indicator performed well for portfolios of large-cap stocks, especially over a short period. Over a «long» period, the effect is somewhat stronger for stocks of small-cap companies. Earlier, [15] argued that for the US market, sentiment had a stronger effect on the performance of stocks of new and small companies.

## Conclusion

Our general conclusion is that sentiment is a significant characteristic in asset pricing in the emerging Russian stock market. For investors, it appears to be insufficient to consider only market ratios, financial metrics, or established trading algorithms to understand how stocks are priced. We consider the Russian market as a proxy for other emerging markets (we study the period up to 2022). A single trend for emerging markets is the active use by investors of communication channels (social networks, messengers, forums) to discuss companies on the Internet. New platforms are emerging—for example, the Reddit platform, which combines the functions of a social network and a forum. Our research showed the increased role of retail investor sentiment in building profitable strategies in emerging stock markets. We obtained this result using AI.

Having examined behavioural bases with a unique dataset for the Russian market (messages on social media), we found that the attention and sentiment of retail investors give rise to a serious anomaly that manifests the inefficiency of an emerging market with a large share of retail investors. We found some similar patterns in different portfolio designs. Over a short period, factors based on stocks’ trading characteristics and factors of sentiment and attention derived from social network communication provided similar results with respect to portfolio excess returns. However, as the time horizon lengthens, the excess returns of the portfolios composed on the basis of sentiment and attention exhibited greater consistency than the momentum portfolios following a U-shaped curve. For the first time we found evidence of nonlinearity for price momentum and behavioural factors. This contributes to already existing puzzles of factor impacts in asset pricing and should encourage studies on the temporal structure of pricing anomalies.

We did not receive empirical support for the hypothesis that sentiment-driven excess returns may be explained by the purchase of low liquid stocks. In contrast, a stronger sentiment effect was observed for large-cap stocks in the short run. The trading strategy of following the crowd when there is a high degree of consensus of investor opinion yielded negative returns in all periods. The lower the liquidity of stocks, the higher the losses. This result is quite surprising, as it contradicts the phenomenon of the small-firm risk premium revealed in many stock markets. We believe that such anomalies could be found in other emerging markets where companies with large market capitalizations and low liquidity operate. Overall, we conclude that discussions and the activity of retail investors affect the pricing of blue chips.

A theoretical implication of our research is that we deepened the understanding of factors forming the risk premium and showed that our Hype indicator is a significant element the risk premium. A practical result of our study is that an exchange of opinions among investors in social networks shaped the mood in the stock market. The first practical implication is that the regulators are advised to monitor and prevent cases of price manipulation arising from discussions on social media. The second practical implication is that an investor can build a profitable trading strategy taking into account the sentiment in social networks. Using sophisticated machine learning techniques and neural networks to identify tones, we built an Hype indicator that allows an investor to compose a stock portfolio that provides an excess return over a period of up to 6 months compared to a portfolio composed on the basis of trading characteristics that only provides an excess return over a period of up to 1 month. We empirically revealed that an investor who trades on market consensus has a high probability of ending up with negative results, especially in the case of low liquid stocks. Trading against consensus is a feasible strategy over a medium to «long» (in relative terms) period of up to 6 months.

The limitations of our study are the following. First, we did not take into account the sentiment of foreign investors, most of whom do not discuss companies in Russian. Also, we did not take into account pictures, emoticons and emoji.

For further research, it would be interesting to employ daily or even intraday data to understand whether pricing factors or buzz in social networks produce an immediate effect. A further improvement in terms of the dataset implies the ranking of messages with respect to the popularity of a blogger and the number of views. For example, it is reasonable to assume that the sentiment of a popular blogger with 100,000 subscribers will carry more weight on retail investors than a chat message with 50 views. With a larger dataset, we could allow for a more detailed classification of messages to test other hypotheses. For example, we could compare positive sentiment for those investors who already hold the stock and for those who plan to buy it, as perhaps the empirical findings for these two groups would be quite different.

## Supporting information

S1 Appendix Descriptive statistics on some emerging stock markets. (DOCX)

S2 Appendix Schematic representation of the research methodology. (DOCX)

S3 Appendix The statistical significance of the nine investment portfolios. (DOCX)

S4 Appendix Portfolios’ monthly excess returns over a medium-term and «long»-term period. (DOCX)

S5 Appendix Robustness check: Portfolio performance for stocks from different market capitalization groups. (DOCX)
