# Supplementary material for: A retail investor in a cobweb of social networks
Source: PLoS One. 2022 Dec 30;17(12):e0276924. doi: 10.1371/journal.pone.0276924 (PMC9803199; doi:10.1371/journal.pone.0276924)
Supplement: S1 Appendix — (DOCX) [file pone.0276924.s001.docx]

**Appendix A. Descriptive statistics on some emerging stock markets**

**Table A.1.**

Descriptive statistics on some emerging stock markets

|  | Market capitalization (USD bln), at the end of the year | Annual trading volume (USD bln) | P/E (at the end of the year) | Annual inflation, % | Minimal key rate (%) | Maximal key rate, % |
| --- | --- | --- | --- | --- | --- | --- |
| 2019 | | | | | | |
| *Russia* | *791.5* | *180.7* | *5.5* | *4.5* | *6.25* | *7.75* |
| Brazil | 1187.4 | 1038.7 | 17.5 | 3.7 | 4.5 | 6.5 |
| Turkey | 185 | 347.6 | 9.2 | 15.2 | 12 | 24 |
| India | 2286.9 | 1290 | 29.2 | 3.7 | 5.15 | 6.5 |
| China | 8515.5 | 18250 | 21.4 | 2.9 | 4.15 | 4.35 |
| S. Africa | 1056.3 | 284.8 | 17.3 | 4.1 | 6.5 | 6.75 |
| Poland | 151.6 | 50.4 | 8.3 | 2.2 | 1.5 | 1.5 |
| Indonesia | 523.3 | 117.9 | 167 | 3 | 5 | 6 |
| 2020 | | | | | | |
| *Russia* | *694.7* | *276.6* | *10.9* | *3.4* | *4.25* | *6.25* |
| Brazil | 988.4 | 1373.6 | 34.4 | 3.2 | 2 | 4.5 |
| Turkey | 237.5 | 868.6 | 16.3 | 12.3 | 8.25 | 17 |
| India | 2595.5 | 1950 | 35.8 | 6.6 | 4 | 5.15 |
| China | 12214.5 | 31580 | 30.8 | 2.4 | 3.85 | 4.15 |
| S. Africa | 1051.5 | 293.8 | 42.7 | 3.3 | 3.5 | 6.5 |
| Poland | 177.5 | 83.4 | 32.4 | 3.4 | 0.1 | 1.5 |
| Indonesia | 496.1 | 131.1 | 26.3 | 1.9 | 3.75 | 5 |
| 2021 | | | | | | |
| *Russia* | *841.9* | *407.3* | *5.8* | *8.4* | *4.25* | *8.5* |
| Brazil | 808.8 | 1550.3 | 7.4 | 8.3 | 2 | 9.25 |
| Turkey | 140.2 | 779.3 | 10.1 | 19.6 | 14 | 19 |
| India | 3575.8 | 2324.3 | 26.1 | 5.1 | 4 | 4 |
| China | 8170 | 43501 | 27 | 1 | 3.8 | 3.85 |
| S. Africa | 1286.9 | 360.8 | 8.9 | 4.6 | 3.5 | 3.75 |
| Poland | 323.3 | 81.8 | 14 | 5.1 | 0.1 | 1.75 |
| Indonesia | 574.5 | 202.1 | 22.6 | 1.6 | 3.5 | 3.75 |

*Source: TR Eikon*
