# Supplementary material for: A retail investor in a cobweb of social networks
Source: PLoS One. 2022 Dec 30;17(12):e0276924. doi: 10.1371/journal.pone.0276924 (PMC9803199; doi:10.1371/journal.pone.0276924)
Supplement: S2 Appendix — (DOCX) [file pone.0276924.s002.docx]

**Appendix B. Schematic representation of the research methodology**

**
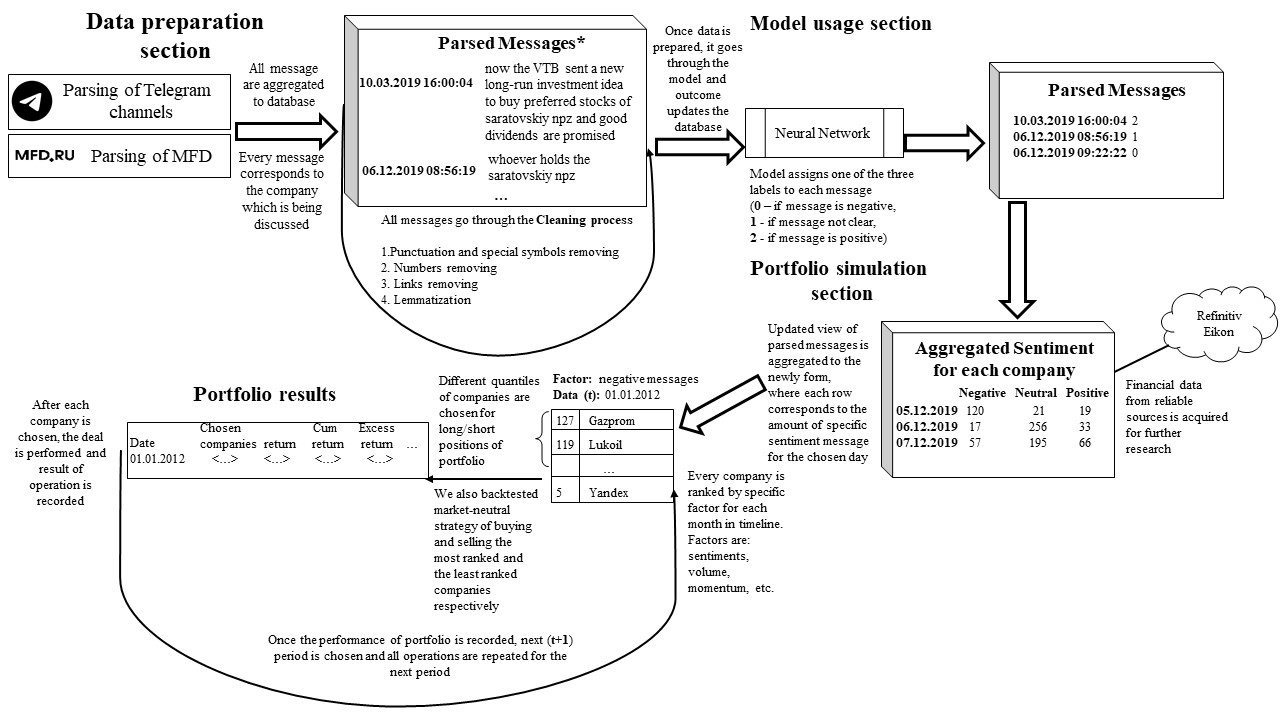
**

**Fig B.1**. **Schematic representation of the research methodology**

*Source: the authors’ methodology*
