# Supplementary material for: A retail investor in a cobweb of social networks
Source: PLoS One. 2022 Dec 30;17(12):e0276924. doi: 10.1371/journal.pone.0276924 (PMC9803199; doi:10.1371/journal.pone.0276924)
Supplement: S3 Appendix — (DOCX) [file pone.0276924.s003.docx]

**Appendix С. The statistical significance of the nine investment portfolios**

**Table С.1.**

The statistical significance of the nine investment portfolios for the 1-0-1 strategy

|  | Alpha | | | | Mean monthly excess return | | | | | Mean monthly return | | | |
| --- | --- | --- | --- | --- | --- | --- | --- | --- | --- | --- | --- | --- | --- |
| Capitalization | all | small | large | mid | all | small | large | mid | all | | small | large | mid |
| 1. Winners | | | | | | | | | | | | | |
| 1.1 The proposed integral indicator of investor sentiment and attention | | | | | | | | | | | | | |
| Hype | 0.39 (1.5) | 0.31 (0.34) | 0.45** (2.12) | -0.26 (-0.93) | 0.58** (2.23) | 0.12 (0.14) | 0.44** (2.17) | -0.28 (-1.04) | 2.17*** (3.03) | | 2.57** (2.09) | 1.74*** (3.04) | 0.76 (1.18) |
| 1.2 Indicators of investor sentiment based on social networks analysis | | | | | | | | | | | | | |
| positive | 0.47 (1.5) | 0.71 (1.05) | 0.45* (1.84) | -0.04 (-0.14) | 0.42 (1.4) | 1.49** (2.1) | 0.22 (0.86) | -0.13 (-0.45) | 2.01*** (3.08) | | 3.93*** (2.81) | 1.52*** (3.03) | 0.91 (1.48) |
| negative | 0.55* (1.89) | 0.3 (0.48) | 0.36 (1.35) | 0.61** (2.02) | 0.66** (2.38) | 1.28* (1.8) | 0.23 (0.88) | 0.53* (1.79) | 2.25*** (3.22) | | 3.73** (2.57) | 1.53*** (2.79) | 1.57** (2.51) |
| consensus | 0.17 (0.73) | -0.18 (-0.26) | 0.17 (0.73) | -0.1 (-0.35) | 0.2 (0.91) | -0.23 (-0.35) | 0.18 (0.82) | -0.06 (-0.22) | 1.8*** (2.75) | | 2.21* (1.95) | 1.48** (2.52) | 0.98 (1.43) |
| 1.3 Indicators of investor attention based on social network analysis | | | | | | | | | | | | | |
| messagesum | 0.64** (2.27) | 0.55 (0.84) | 0.43* (1.98) | 0.2 (0.69) | 0.57** (2.12) | 1.5** (2.08) | 0.3 (1.41) | 0.2 (0.72) | 2.17*** (3.41) | | 3.94*** (2.72) | 1.6*** (3.03) | 1.24* (1.88) |
| relative_attention | 0.53* (1.87) | 0.09 (0.12) | 0.43* (1.98) | 0.24 (0.81) | 0.29 (1.03) | 0.3 (0.42) | 0.3 (1.41) | 0.26 (0.93) | 1.89*** (3.26) | | 2.75** (2.19) | 1.6*** (3.03) | 1.3* (1.93) |
| 1.4 Indicators of investor attention based on trading characteristics | | | | | | | | | | | | | |
| price momentum | 0.56* (1.95) | 0.58 (0.88) | 0.31 (1.04) | 0.56** (2.09) | 0.65** (2.39) | 1.6** (2.17) | 0.13 (0.42) | 0.56** (2.12) | 2.25*** (3.24) | | 4.05*** (2.74) | 1.43** (2.63) | 1.6** (2.46) |
| volatility | 0.4 (1.31) | -0.03 (-0.05) | 0.23 (0.95) | 0.25 (0.88) | 0.48 (1.63) | 0.64 (0.95) | 0.07 (0.3) | 0.16 (0.59) | 2.07*** (2.99) | | 3.09** (2.28) | 1.37** (2.6) | 1.2* (1.96) |
| volume | 0.36 (1.2) | 0.65 (0.8) | 0.32 (1.26) | 0.23 (0.77) | 0.23 (0.82) | 0.3 (0.38) | 0.18 (0.75) | 0.35 (1.19) | 1.83*** (2.93) | | 2.74** (2.46) | 1.49*** (2.75) | 1.39* (1.91) |
| 2. Losers | | | | | | | | | | | | | |
| Hype | -0.19 (-0.66) | 0.11 (0.13) | -0.43** (-2.0) | 0.25 (0.86) | -0.37 (-1.34) | -0.46 (-0.55) | -0.42** (-2.04) | 0.18 (0.64) | 1.23** (2.06) | | 1.98* (1.83) | 0.88 (1.51) | 1.22* (1.95) |
| consensus | -0.15 (-0.62) | 0.16 (0.23) | -0.19 (-0.79) | -0.02 (-0.08) | -0.21 (-0.9) | -0.0 (-0.0) | -0.19 (-0.85) | -0.14 (-0.48) | 1.39** (2.21) | | 2.44** (2.21) | 1.11* (1.9) | 0.9 (1.5) |
| 3. WML | | | | | | | | | | | | | |
| Hype | 0.58 (1.09) | 0.2 (0.12) | 0.89** (2.06) | -0.51 (-0.91) | -0.65 (-0.95) | -1.86 (-1.09) | -0.44 (-0.64) | -1.5* (-1.9) | 0.95* (1.81) | | 0.58 (0.38) | 0.86** (2.1) | -0.46 (-0.85) |
| consensus | 0.32 (0.68) | -0.34 (-0.25) | 0.36 (0.76) | -0.08 (-0.14) | -1.19 (-1.64) | -2.67* (-1.69) | -0.93 (-1.33) | -0.97 (-1.29) | 0.41 (0.91) | | -0.23 (-0.18) | 0.37 (0.83) | 0.07 (0.13) |

*Source: the authors’ calculations*

*Note: The following winner portfolios of all stocks in the sample produce a significant and positive mean monthly excess return: based on the Hype indicator, negative sentiment, the total number of messages, and relative attention to a company. With the Hype indicator, it is possible to design a portfolio with a positive and significant alpha for large-cap stocks. No portfolio of small-cap stocks produces a significant alpha. The loser portfolio of large-cap stocks composed based on the Hype indicator produces a negative and significant alpha and mean monthly excess return. In contrast, the WML portfolio of large-cap stocks based on the Hype indicator generates a positive and significant alpha. The WML portfolio of small-cap stocks based on the crowd’s consensus generates a negative and significant mean monthly return.*

**Table С.2.**

The statistical significance of the nine investment portfolios for the 3-0-3 strategy

|  | Alpha | | | | Mean monthly excess return | | | | Mean monthly return | | | |
| --- | --- | --- | --- | --- | --- | --- | --- | --- | --- | --- | --- | --- |
| Capitalization | all | small | large | mid | all | small | large | mid | all | small | large | mid |
| 1. Winners | | | | | | | | | | | | |
| HYPE | -0.09 (-0.23) | 0.02 (0.02) | -0.68*** (-3.27) | -0.013 (-0.05) | 0.073 (0.22) | -0.677 (-0.78) | -0.227 (-1.04) | 0.237 (0.87) | 1.847** (2.68) | 1.957* (1.73) | 1.177* (1.96) | 1.47** (2.09) |
| consensus | -0.36 (-1.16) | -0.643 (-1.07) | 0.05 (0.19) | -0.743* (-1.72) | -1.033*** (-3.14) | -2.207** (-2.7) | -0.083 (-0.39) | -0.983** (-2.48) | 0.737* (1.73) | 0.423 (0.63) | 1.32*** (2.96) | 0.253 (0.43) |
| 2. Losers | | | | | | | | | | | | |
| HYPE | 0.2 (0.52) | 0.37 (0.41) | 0.63*** (3.2) | 0.06 (0.2) | 0.143 (0.46) | 0.967 (1.18) | 0.237 (1.17) | -0.303 (-0.98) | 1.917*** (3.09) | 3.597** (2.38) | 1.64*** (4.66) | 0.93* (1.99) |
| consensus | 0.57 (1.34) | 1.1 (1.09) | -0.03 (-0.11) | 0.697 (1.68) | 0.82** (2.3) | 1.427 (1.62) | 0.097 (0.45) | 0.857** (2.3) | 2.593*** (3.59) | 4.06** (2.75) | 1.503*** (2.88) | 2.09*** (2.91) |
| 3. WML | | | | | | | | | | | | |
| HYPE | -0.29 (-0.38) | -0.35 (-0.19) | -1.31*** (-3.26) | -0.073 (-0.13) | -1.843** (-2.33) | -4.27* (-1.91) | -1.867*** (-5.05) | -0.693 (-1.22) | -0.07 (-0.11) | -1.64 (-0.98) | -0.463 (-1.11) | 0.54 (0.95) |
| consensus | -0.933 (-1.41) | -1.74 (-1.22) | 0.077 (0.15) | -1.44* (-1.72) | -3.627*** (-3.63) | -6.263*** (-2.85) | -1.587** (-2.37) | -3.073*** (-2.97) | -1.857*** (-3.05) | -3.633** (-2.5) | -0.183 (-0.42) | -1.84** (-2.42) |

*Source: the authors’ calculations*

*Note: We observe the overreaction effect over a period of 3 months. The winner portfolios composed based on the Hype indicator and the crowd’s consensus produce negative alphas virtually for all market capitalisation groups. The loser portfolio composed based on the Hype indicator produces a positive and significant alpha, while the WML portfolio composed based on the Hype indicator generates a negative and significant alpha. The lowest mean monthly excess return is observed for the WML portfolio composed based on the crowd’s consensus*
