# Supplementary material for: A retail investor in a cobweb of social networks
Source: PLoS One. 2022 Dec 30;17(12):e0276924. doi: 10.1371/journal.pone.0276924 (PMC9803199; doi:10.1371/journal.pone.0276924)
Supplement: S4 Appendix — (DOCX) [file pone.0276924.s004.docx]

**Appendix D. Portfolios’ monthly excess returns over a medium-term and «long»-term period**


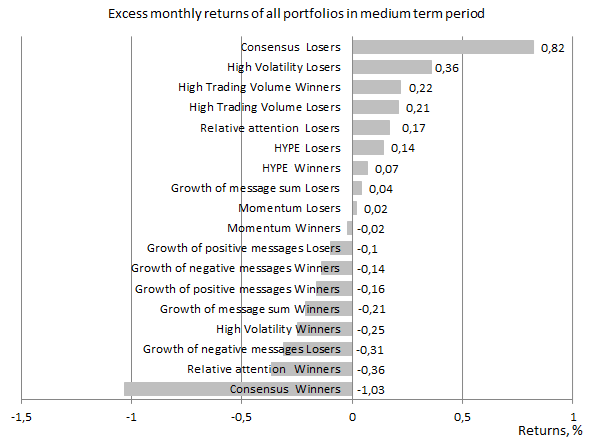


**Fig D.1**. **Excess returns on each portfolio over a medium-term period**

*Source: the authors’ calculations*


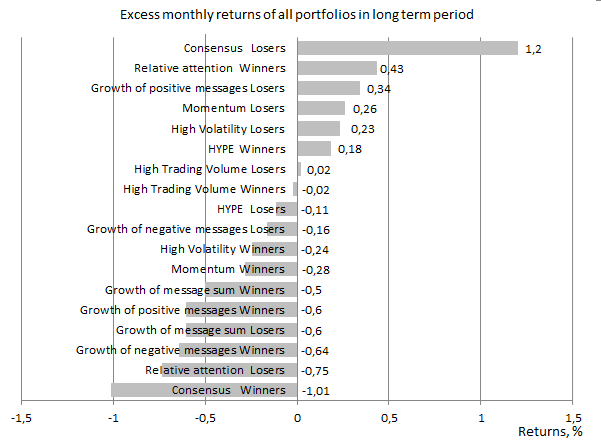


**Fig D.2. Excess returns on each portfolio over a «long»-term period**

*Source: the authors’ calculations*
