# Supplementary material for: A retail investor in a cobweb of social networks
Source: PLoS One. 2022 Dec 30;17(12):e0276924. doi: 10.1371/journal.pone.0276924 (PMC9803199; doi:10.1371/journal.pone.0276924)
Supplement: S5 Appendix — (DOCX) [file pone.0276924.s005.docx]

**Appendix E. Robustness check: portfolio performance for stocks from different market capitalization groups**


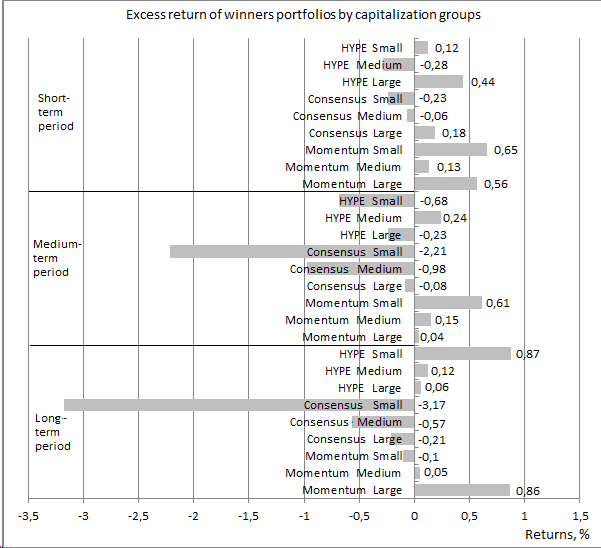


**Fig E.1. Excess returns for the winner portfolios of small-cap, mid-cap, and large-cap stocks**

*Source: the authors’ calculations*

**Table E.1**

Statistical significance of mean monthly excess returns on portfolios of stocks within the 40% subsample

|  | Mean monthly return | | | |
| --- | --- | --- | --- | --- |
| Capitalization | all | small | large | mid |
| 1.1 The proposed integral indicator of investor sentiment and attention | | | | |
| Hype | 2.0** (2.46) | 3.69** (2.0) | 1.73*** (2.97) | 1.16 (1.47) |
| 3.2 Indicators of investor sentiment based on social network analysis | | | | |
| positive | 1.15 (1.61) | 2.86* (1.84) | 1.22* (1.92) | 0.51 (0.71) |
| negative | 1.69** (2.16) | 4.67** (2.36) | 1.51** (2.38) | 0.76 (1.02) |
| consensus | 1.96** (2.53) | 1.72 (1.59) | 1.56*** (2.8) | 0.88 (1.35) |
| 3.3 Indicators of investor attention based on social network analysis | | | | |
| messagesum | 1.71** (2.37) | 2.75* (1.8) | 1.64*** (2.82) | 0.46 (0.66) |
| relative_ attention | 1.74** (2.42) | 2.52* (1.7) | 1.7*** (2.93) | 0.59 (0.77) |
| 3.4 Indicators of investor attention based on trading characteristics | | | | |
| price momentum | 1.15** (2.13) | 2.96* (1.85) | 0.7 (1.18) | 1.21** (2.29) |
| volatility | 2.08** (2.56) | 4.13** (2.14) | 1.38*** (2.79) | 0.94 (1.43) |
| volume | 1.44*** (2.65) | 1.71 (1.66) | 1.79*** (3.5) | 0.93 (1.22) |

*Source: the authors’ calculations*

*Note: Virtually for all portfolios (except for the group of mid-cap stocks)*, *mean monthly returns are positive and significant. The best performing portfolios of all stocks in the sample are those based on the Hype indicator and volatility. Within the group small-cap stocks, the best performing portfolios are those based on negative investor sentiment and volatility. Within the group of large-cap stocks the best performing portfolios are those based on the Hype indicator and trading volume.*
